# Characterization of individuals with skeletal dysplasia at a referral center in Brazil

**DOI:** 10.1007/s12687-026-00920-9

**Published:** 2026-07-07

**Authors:** J. G. C. Meira, M. P. Migliavacca, A. X. Acosta

**Affiliations:** 1 Department of Life Sciences, Bahia State University, Salvador, BA Brazil; 2 Hospital Israelita Albert Einstein, São Paulo, SP Brazil; 3 Department of Pediatrics, Faculty of Medicine of Bahia, Federal University of Bahia, Salvador, BA Brazil; 4 Medical Genetics Service, Complexo Hospitalar Universitário Professor Edgard Santos, Salvador, BA Brazil

**Keywords:** Skeletal dysplasia, Population characteristics, Phenotype, Ontologies as topic, Orphan diseases

## Abstract

Skeletal dysplasias are rare genetic disorders affecting bone and cartilage, often causing disproportionate short stature and multisystem involvement. In Brazil, limited data challenge diagnosis and management. To describe the clinical and sociodemographic profile of individuals with suspected skeletal dysplasia, without confirmed etiological diagnosis, evaluated at a university hospital in Salvador, Bahia, Brazil, and referred for genomic sequencing through the Rare Genomes Project. Observational, cross-sectional study including 90 individuals evaluated at Hospital Professor Edgard Santos, that is part of Federal University of Bahia, between December 2020 and May 2023. All patients were evaluated by a medical geneticist, and clinical data were extracted from medical records and standardized using Human Phenotype Ontology (HPO) terms. Most participants (71%) were from countryside of Bahia, 68% were mixed-race, with balanced sex distribution. The mean age was 11.4 years. Consanguinity was reported in 28% and family recurrence in 34% of cases. Among the 15 subgroups listed, the most frequent was “Skeletal dysplasia with decreased bone density” (43.3%). A total of 299 distinct HPO terms reflected high phenotypic variability. This study highlights the clinical heterogeneity of skeletal dysplasia and the importance of a specialized evaluation by a clinical geneticist enabling standardized phenotyping combined with genomic tools to improve diagnosis and public health care.

## Introduction

Rare diseases (RD) are individually rare but collectively affect a significant proportion of the population, and approximately 71.9% have a genetic basis that requires specific diagnostic testing (Nguengang Wakap et al. 2020). Among them, hereditary skeletal disorders (HSD), include skeletal dysplasias (SD), osteodystrophies, and dysostoses. SD, or osteochondrodysplasias (OCD), are progressive disorders caused by intrinsic bone and cartilage defects; osteodystrophies involve metabolic alterations and dysostoses result from abnormal bone formation with a static phenotype (Hall 2002; Offiah and Hall 2003). The 2023 Nosology of Genetic Skeletal Disorders lists 771 conditions in 41 categories, linked to 552 genes (Unger et al. 2023).

With a prevalence of 2.3–3.2 per 10,000 (Orioli, Castillat, and Barbosa-Neto 1986; Stevenson et al. 2012; Stoll et al. 1989) SD often cause short stature and multisystem involvement, ranging from mild to lethal forms (Kolambage et al. 2023; Legare and Basel 2023). Diagnosis is crucial but challenging, especially in public health systems. To expand diagnostic access, the Rare Genomes Project (PGR) was launched in 2020 as a partnership between the Hospital Israelita Albert Einstein (HIAE) and the Brazilian Ministry of Health, aiming to enable RD diagnosis within the public healthcare system (Coelho et al. 2022). Technologies like Next-Generation Sequencing (NGS) have advanced our understanding of their genetic basis (Méndez-Vidal et al. 2025; Mortier et al. 2019).

Given the limited epidemiological data on SD in Brazil, this study aims to characterize the clinical and sociodemographic profiles of individuals with suspected SD evaluated at University Hospital Professor Edgard Santos (HUPES), Salvador, Bahia, Brazil.

## Methods

This observational, descriptive, cross-sectional study included a convenience sample of 90 individuals with suspected SD and no defined diagnosis evaluated from December 2020 to May 2023, at the Medical Genetics Service outpatient clinic (SGM) of HUPES, Federal University of Bahia (UFBA), a national RD referral center since 2019 (Cavalcanti et al. 2020).

All participants were enrolled in the PGR, provided informed consent, had clinical data recorded in PhenoTips^®^, and provided samples for whole genome sequencing (WGS), currently under analysis (Coelho et al. 2022; Girdea et al. 2013). Patients with obvious clinical diagnoses, e.g., achondroplasia (ACH) were excluded to focus on undiagnosed SD cases and optimize WGS use.

Clinical and demographic data, family and medical history, and phenotypic features (coded using Human Phenotype Ontology terms (HPO): https://hpo.jax.org/) were extracted from electronic medical records and PhenoTips^®^. Patients were categorized into 15 clinical HSD subtypes based on predominant phenotypes (Table 1). Data were compiled in Microsoft Excel (version 2605) and analyzed descriptively using frequencies, means, medians, and standard deviations.

## Results

The sociodemographic characteristics, HSD group distribution, and age at symptom onset of the 90 individuals who fulfilled the HSD cohort criteria are presented in Table 1.

**Table 1:** Sociodemographic profile, HSD ^a^ group distribution, and age at symptom onset of individuals from HUPES ^b^ and enrolled in the PGR ^c^ (Dec 2020–May 2023).

	Variables	N	%
Sex	Male	45	50%
	Female	45	50%
Race/Color	White	19	21%
	Brown (Parda)	61	68%
	Black	10	11%
Age group (years)	0–4	22	24%
	5–9	25	28%
	10–14	24	27%
	> 15	19	21%
Place of Birth	Salvador-BA^d^	22	24.5%
	Metropolitan Region of Salvador-BA^e^	25	28%
	Other macroregions of BA	64	71%
	Other states	4	4.5%
Origin	Salvador-BA	15	17%
	Metropolitan Region of Salvador-BA	23	26%
	Other macroregions of BA	75	83%
HSD Subgroup	SD^f^ with decreased bone density	39	43.3%
	SD with metaphyseal predominance	8	8.9%
	Disostoses	6	6.7%
	SD with epiphyseal predominance	5	5.6%
	SD with osteolytic lesions	5	5.6%
	SD with diaphyseal predominance	4	4.4%
	SD with sclerosing manifestations	4	4.4%
	SD with distal segment predominance	4	4.4%
	SD with epiphyseal spine predominance	3	3.3%
	SD with epi-metaphyseal spine predominance	1	1.1%
	SD with dislocations	1	1.1%
	SD *FGFR*	1	1.1%
	SD primordial dwarfism	1	1.1%
	SD skeletal ciliopathies	1	1.1%
	Others	7	7.8%
Age at symptom onset	Birth	35	38.9%
	< 1 year	19	21.1%
	1–2 years	19	21.1%
	3–6 years	9	10.0%
	7–10 years	4	4.4%
	11–18 years	4	4.4%

^a^*HSD* Hereditary Skeletal Dysplasias, ^b^*HUPES* University Hospital Professor Edgard Santos, ^c^*PGR* Genomas Raros Project, ^d^*BA* State of Bahia, ^e^The Metropolitan Region of Salvador comprises 13 municipalities including Salvador, ^f^*SD* Skeletal Dysplasias

The cohort showed balanced sex distribution and 68% mixed-race individuals, aged 4 months to 53 years (mean: 11.4; median: 9; IQR: 4.75–14). The most frequent age group was 5–9 years. Most participants were born (71%) and resided (83%) in countryside Bahia; 28% were born in the Salvador Metropolitan Region, and 4% from other states. Age at symptom onset ranged from birth to 18 years, with the highest proportion of cases at birth (38.9%), followed by onset within the first year of life (21.1%) and between 1 and 2 years (21.1%). Fewer cases presented between 3 and 6 years (10.0%), 7–10 years (4.4%), and 11–18 years (4.4%) (Table 1).

Birth weight ranged from 1,200 g to 4,140 g (mean 2.975 g; median 2.915 g; IQR 2.678–3.848 g) with 17% with low birth weight (< 2,500 g) and 6% preterm. Parental consanguinity and familial recurrence occurred in 28% and 34%, respectively. Maternal age at delivery ranged 15–42 years (mean: 25.71; median: 24; IQR: 20–30.5), with 17.6% ≥35 years. Paternal age ranged 18–59 years (mean: 31; median: 30.5; IQR: 23.25–36.75), unknown in 15.5%, with 19.7% ≥40 years.

**Fig. 1:**
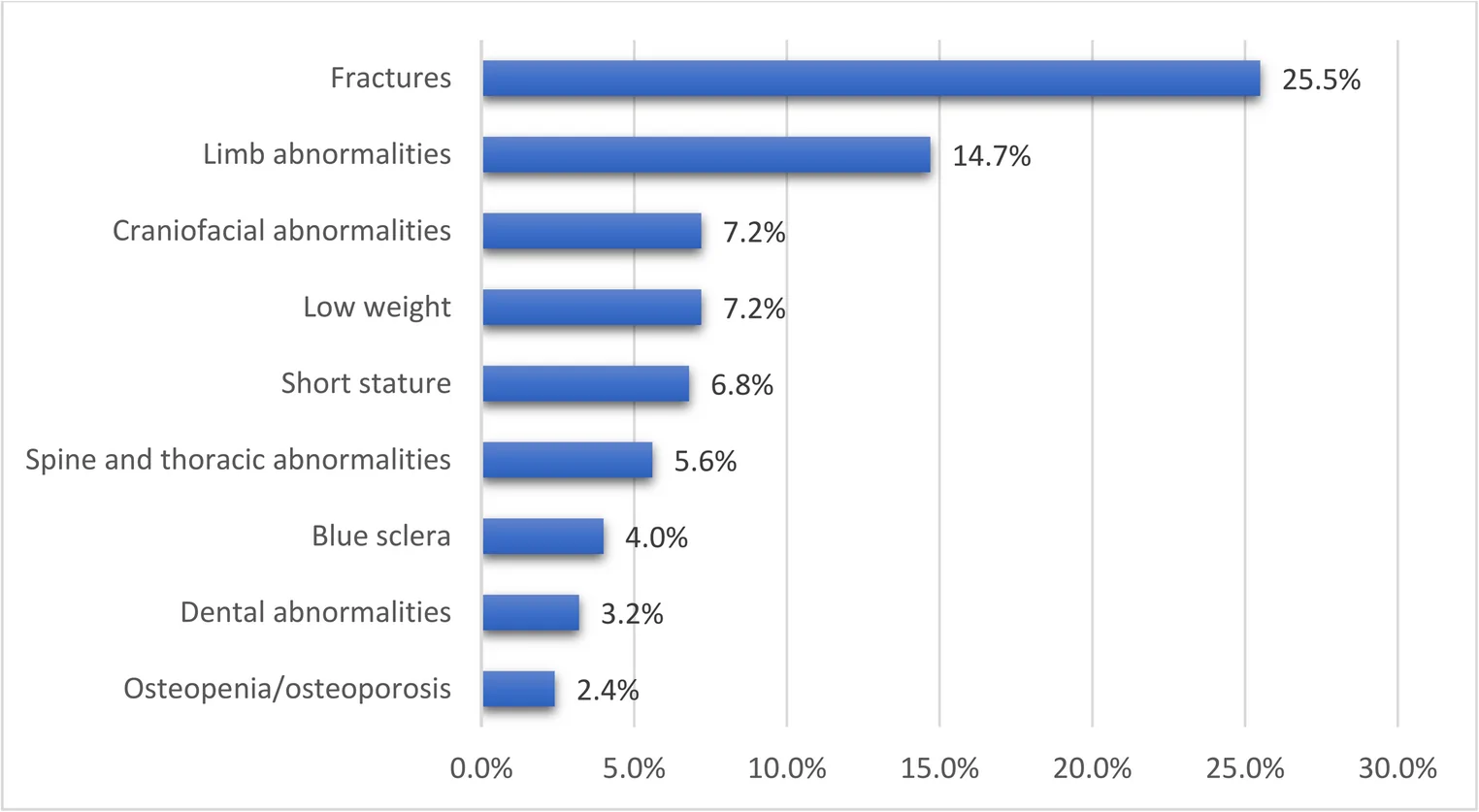
Grouped distribution of frequent HPO ^a^ terms in the “Decreased Bone Density” subgroup from HUPES ^b^ and enrolled in the PGR ^c^ (Dec 2020–May 2023). ^a^ *HPO* Human Phenotype Ontology, ^b^ *HUPES* University Hospital Professor Edgard Santos, ^c^ *PGR*: Genomas Raros Project

The most frequent HSD subgroup was “Decreased Bone Density” (43.3%) (Table 1). Among 39 individuals of this group, 98 HPO terms were cited 251 times, highlighting high clinical variability. Similar terms were grouped for analysis (Fig. 1).

Figure 1 shows key HPO terms in the “Decreased Bone Density” group. Fractures were most common (25.5%), followed by several descriptions of limb abnormalities (14.7%), low weight (7.2%), short stature (6.8%), spine and thoracic anomalies (5.6%), blue sclera (4%), dental abnormalities (3.2%), and osteopenia/osteoporosis (2.4%). Bone pain (0.8%), tall stature (0.8%), and obesity (2.4%) were rare. Thirteen distinct craniofacial anomalies were identified in the cohort (7.2%). Triangular face was the most frequently reported facial dysmorphism (2%), whereas the other craniofacial features occurred at lower frequencies (Fig. 1). Perinatal complications (7.6%) and cesarean delivery (6,4%) were common. Neurological abnormalities (seizures, developmental delays, neuropathy, gait disorder) accounted for 2% of citations. Rare non-skeletal features (e.g., such retinal detachment, patent foramen ovale, respiratory discomfort and skin hyperpigmentation) were cited once each. The generic term *Skeletal Dysplasia* appeared only three times.

**Fig. 2:**
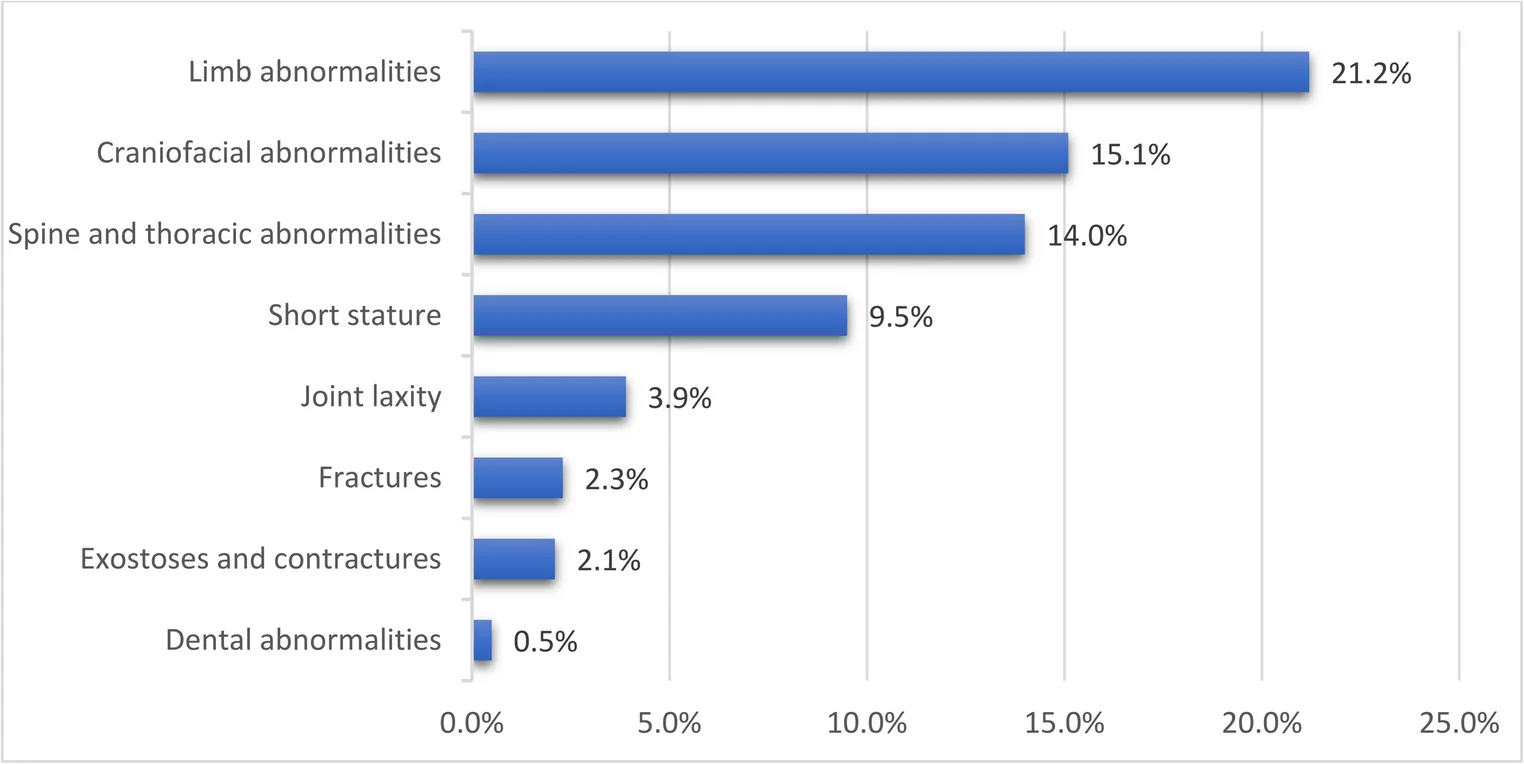
Distribution of osteomuscular and craniofacial HPO ^a^ terms in HSD ^b^ subgroups (excluding “Decreased Bone Density”) from HUPES ^c^ and enrolled in the PGR ^d^ (Dec 2020–May 2023). ^a^ *HPO* Human Phenotype Ontology, ^b^ *HSD* Hereditary Skeletal Dysplasias, ^c^ *HUPES* University Hospital Professor Edgard Santos, ^d^ *PGR* Genomas Raros Project

Excluding the “Decreased Bone Density” group, the remaining HSD groups (cited on Table 1) showed 570 citations of 288 HPO terms described 51 individuals. Osteomuscular and craniofacial phenotypes accounted for 68.6%, mainly limb anomalies (21.2%), followed by craniofacial (15.1%), spine/thorax anomalies (14%), and short stature (9.5%). Joint laxity, fractures, exostoses, contractures, and dental abnormalities were less common (Fig. 2). Phenotypes from other systems represented 17.7% of citations, mainly neurological (4.0%), ophthalmological (3.9%) and auditory (3.0%). Dermatological (1.8%), endocrinological (1.8%), genitourinary (1.1%), and cardiovascular (0.9%) were less common. Perinatal complications (4.6%) and cesarean delivery (4.2%) were also frequent. Non-specific terms like *Skeletal Dysplasia* and *Multiple Skeletal Anomalies* represented 2.5%, while low birth weight accounted for 2.3%. Rare terms (*digital pads*, *disproportionate tall stature*, *laryngomalacia*,* supernumerary nipple*) appeared once each; *dorsalgia*, twice.

## Discussion

This study examined the clinical and sociodemographic profile of 90 individuals with HSD, aged 4 months to 53 years, evaluated at SGM-HUPES in Bahia, Brazil, addressing the scarcity of national data.

Most participants were born in the countryside of Bahia (71%) and lived outside Salvador (83%). Despite this, the centralization of reference services in the capital and Bahia’s vast territory may limit access for individuals in remote areas, suggesting possible underreporting.

The cohort had balanced sex distribution, 68% self-identified as mixed-race, and a mean age of 11.4 years. The sex ratio aligns with Latin America (Barbosa-Buck et al. 2012), though other studies report slight male predominance in southern India (1.4:1) (Nampoothiri et al. 2014) and Turkey (1.28:1) (Kurt-Sukur et al. 2015), and female predominance in Sri Lanka (Kolambage et al. 2023). These patterns reflect SD inheritance profiles: autosomal inheritance (dominant or recessive) is predominant, while X-linked forms are rare. A 2023 review reported 4.4% of SDs as X-linked, compared to 44.1% autosomal dominant and 51.5% autosomal recessive (AR) (Unger et al. 2023).

Mean maternal age was 25.71 years, with 17.6% aged ≥ 35— similar to values in Argentina and Utah (27.1) and Latin America (26.4) (Barbosa-Buck et al. 2012; Duarte et al. 2019; Stevenson et al. 2012). Although definitions vary, most studies define advanced paternal age as ≥ 40 years (Neeser et al. 2023). Here, mean paternal age was 31 years, with 19.7% over 40 — comparable to findings by Stevenson et al. (30.5) and Barbosa-Buck et al. (31.2), who linked older paternal age to higher SD risk (Barbosa-Buck et al. 2012; Stevenson et al. 2012). This may be explained by the high frequency of thanatophoric dysplasia (TD) and ACH in those studies—conditions caused by *FGFR3* mutations, which are strongly linked to advanced paternal age (Moura et al. 2024). In contrast, the present study excluded lethal forms such as TD, as well as cases of ACH.

Regarding neonatal findings, 38.9% presented evident phenotype at birth, and symptom onset ranged from 0 to 18 years. Additionally, 17% had low birth weight (mean: 2,975 g) and 6% were premature, lower than in studies including lethal or stillbirth cases, which reported prematurity proportion of 34–38.4% and lower birth weights (2,491–2,550 g) (Barbosa-Buck et al. 2012; Duarte et al. 2019).

The identification of consanguinity and family recurrence of SD is essential to guiding the diagnostic approach. Consanguinity was observed in 28% and familial recurrence in 34%, consistent with Utah data, with 31% of family recurrence (Stevenson et al. 2012). Consanguinity frequency varies worldwide: 13.6% in southern India (Nampoothiri et al. 2014), 5,4% in Latin America (Barbosa-Buck et al. 2012) and 53% in Turkey (Kurt-Sukur et al. 2015) reflecting regional and cultural differences. The high proportion here may reflect AR later-onset SD forms linked to consanguinity.

The most frequent subgroup involved suspected low bone density (43.3%), supporting previous reports: 23.4% with Osteogenesis Imperfecta (OI) in Barbosa-Buck et al., 78% with ACH/OI/TD in Duarte et al. and 60.6% with OI in Kolambage (Barbosa-Buck et al. 2012; Duarte et al. 2019; Kolambage et al. 2023). In Utah and Sri Lanka, OI was also highly prevalent (Kurt-Sukur et al. 2015; Stevenson et al. 2012).

A total of 299 distinct HPO terms were used, indicating high clinical variability. In the “Decreased Bone Density” group, 98 terms were cited 251 times, mainly reflecting classical OI features. On the other hand, *bone pain* was rarely mentioned (*n* = 2), suggesting underreporting. In the remaining subgroups (51 individuals), 288 HPO terms were cited 570 times, highlighting limb, spine/thorax anomalies, short stature, and 42 craniofacial terms. Fractures were rare (2.3%), but multisystemic involvement was common.

Perinatal complications (4.6%–7%) and cesarean deliveries (4.2%–6.4%) were recurrent across subgroups, suggesting prenatal anomalies or intrauterine fractures not systematically investigated in this sample, which may explain the high frequency of symptoms at birth.

Only 22 of the 98 HPO terms for suspected OI matched those listed on the HPO platform for “Osteogenesis Imperfecta,” (https://hpo.jax.org/browse/disease/ORPHA:666) and only 14 of the 288 terms used in other subgroups matched those under “Osteochondrodysplasia” (https://hpo.jax.org/browse/disease/OMIM:184260). These findings highlight the phenotypic variability in HSD and a lack of systematized correlations between HPO terms and clinical presentation.

The clinical presentation of the syndromes included in this review involves manifestations across multiple organ systems. Although the skeletal phenotype is the predominant and defining feature, extra-skeletal comorbidities are also part of the phenotypic spectrum, with frequencies ranging from 0.9% to 4.0%. Neurological involvement was the most frequent, including developmental delay affecting motor, speech, and/or cognitive domains. Ophthalmological (3.9%) and auditory (3.0%) findings may contribute to developmental delay, particularly in communication and cognitive domains, while skeletal abnormalities may further contribute to motor delay.

## Conclusion

This study provides new insights into the clinical and demographic profile of individuals with HSD in Bahia, Brazil—mostly from countryside areas, with significant representation from the capital. The cohort showed balanced sex distribution, high consanguinity (28%) and familial recurrence (34%), suggesting strong genetic influence.

International comparisons show similar sex distribution, parental age, and birth weight, but differences in prematurity and consanguinity proportions, reflecting differences in population structure and cultural practices. Assessment of family history remains essential for diagnostic evaluation and genetic counseling.

Clinical and dysmorphological evaluations by medical geneticists enabled detailed phenotyping and correlation with potential molecular findings. Most cases exhibited low bone density indicative of OI, confirming this subgroup’s significance.

HPO analysis revealed substantial phenotypic diversity, highlighting diagnostic challenges and supporting integrated strategies that combine clinical assessment, standardized terminology, and genomic analysis.

Such strategies are crucial for early recognition, timely interventions, and effective counseling. Despite being a retrospective, single-center study, by excluding cases with a classical clinical diagnosis, it represents the first regional analysis focused on individuals with suspected SD without confirmed etiological diagnosis. It lays a foundation for a national HSD registry in Brazil, in collaboration with other research groups.

## Data Availability

No datasets were generated or analysed during the current study.

## References

[CR1] Barbosa-Buck CO, Orioli IM, da Graça Dutra M, Lopez-Camelo J, Castilla EE, Cavalcanti DP (2012) Clinical epidemiology of skeletal dysplasias in South America. Am J Med Genet A 158A(5):1038–1045. 10.1002/ajmg.a.35246 22407836

[CR2] Cavalcanti DP, Fano V, Mellado C, Lacarrubba-Flores MDJ, Silveira C, Silveira KC et al (2020) Skeletal dysplasias in Latin America. Am J Med Genet C 184:986–995. 10.1002/ajmg.c.31861 PMC9827228 33219737

[CR3] Coelho AVC, Mascaro-Cordeiro B, Lucon DR, Nóbrega MS, Reis RS, de Alexandre RB et al (2022) The Brazilian Rare Genomes Project: Validation of Whole Genome Sequencing for Rare Diseases Diagnosis. Front Mol Biosci 9. 10.3389/fmolb.2022.821582 PMC9108541 35586190

[CR4] Duarte SP, Rocha ME, Bidondo MP, Liascovich R, Barbero P, Groisman B (2019) Bone dysplasias in 1.6 million births in Argentina. Eur J Med Genet 62(12). 10.1016/j.ejmg.2018.12.008 30572171

[CR5] Girdea M, Dumitriu S, Fiume M, Bowdin S, Boycott KM, Chénier S et al (2013) PhenoTips: Patient phenotyping software for clinical and research use. Hum Mutat 34(8):1057–1065. 10.1002/humu.22347 23636887

[CR6] Hall CM (2002) International nosology and classification of constitutional disorders of bone (2001). Am J Med Genet 113(1):65–77. 10.1002/ajmg.10828 12400068

[CR7] Kolambage YD, Walpita YN, Liyanage UA, Dayaratne BMKDR, Dissanayake VHW (2023) The burden of hospital admissions for skeletal dysplasias in Sri Lanka: a population-based study. Orphanet J Rare Dis 18(1). 10.1186/s13023-023-02884-2 PMC10485930 37684696

[CR8] Kurt-Sukur ED, Simsek-Kiper PO, Utine GE, Boduroglu K, Alanay Y (2015) Experience of a skeletal dysplasia registry in Turkey: A five-years retrospective analysis. Am J Med Genet A 167(9):2065–2074. 10.1002/ajmg.a.37122 25931420

[CR9] Legare JM, Basel D (2023) What the pediatric endocrinologist needs to know about skeletal dysplasia, a primer. Front Pediatr 11. 10.3389/fped.2023.1229666 PMC10477785 37675393

[CR10] Méndez-Vidal C, Bravo-Gil N, Pérez-Florido J, Marcos-Luque I, Fernández RM, Fernández-Rueda JL et al (2025) A genomic strategy for precision medicine in rare diseases: integrating customized algorithms into clinical practice. J Transl Med 23(1). 10.1186/s12967-025-06069-2 PMC11748347 39833864

[CR11] Mortier GR, Cohn DH, Cormier-Daire V, Hall C, Krakow D, Mundlos S et al (2019) Nosology and classification of genetic skeletal disorders: 2019 revision. Am J Med Genet A 179(12):2393–2419. 10.1002/ajmg.a.61366 31633310

[CR12] Moura S, Hartl I, Brumovska V, Calabrese PP, Yasari A, Striedner Y et al (2024) Exploring FGFR3 Mutations in the Male Germline: Implications for Clonal Germline Expansions and Paternal Age-Related Dysplasias. Genome Biol Evol 16(2). 10.1093/gbe/evae015 PMC10898338 38411226

[CR13] Nampoothiri S, Yesodharan D, Sainulabdin G, Narayanan D, Padmanabhan L, Girisha KM et al (2014) Eight years experience from a skeletal dysplasia referral center in a tertiary hospital in Southern India: A model for the diagnosis and treatment of rare diseases in a developing country. Am J Med Genet A 164(9):2317–2323. 10.1002/ajmg.a.36668 25044831

[CR14] Neeser NB, Martani A, De Clercq E, De Geyter C, Vulliemoz N, Elger BS et al (2023) Building a family at advanced parental age: a systematic review on the risks and opportunities for parents and their offspring. Hum Reprod Open. 10.1093/hropen/hoad042 38045093 PMC10692762

[CR15] Offiah AC, Hall CM (2003) Radiological diagnosis of the constitutional disorders of bone. As easy as A, B, C? Pediatr Radiol 33:153–161. 10.1007/s00247-002-0855-8 12612812

[CR16] Orioli IM, Castilla EE, Barbosa-Neto JG (1986) The birth prevalence rates for the skeletal dysplasias. J Med Genet 23. 10.1136/jmg.23.4.328 PMC1049699 3746832

[CR17] Stevenson DA, Carey JC, Byrne JLB, Srisukhumbowornchai S, Feldkamp ML (2012) Analysis of skeletal dysplasias in the Utah population. Am J Med Genet A 158A(5):1046–1054. 10.1002/ajmg.a.35327 22461456

[CR18] Stoll C, Don B, Roth MP, Alembik Y (1989) Birth prevalence rates dysplasias of skeletal. Clin Genet 35. 10.1111/j.1399-0004.1989.tb02912.x 2785882

[CR19] Unger S, Ferreira CR, Mortier GR, Ali H, Bertola DR, Calder A et al (2023) Nosology of genetic skeletal disorders: 2023 revision. Am J Med Genet A 191(5):1164–1209. 10.1002/ajmg.a.63132 36779427 PMC10081954

[CR20] Wakap NS, Lambert DM, Olry A, Rodwell C, Gueydan C, Lanneau V et al (2020) Estimating cumulative point prevalence of rare diseases: analysis of the Orphanet database. Eur J Hum Genet 28(2):165–173. 10.1038/s41431-019-0508-0 31527858 PMC6974615

